# Endothelial NO Synthase Gene Polymorphisms and Risk of Ischemic Stroke in Asian Population: A Meta-Analysis

**DOI:** 10.1371/journal.pone.0060472

**Published:** 2013-03-27

**Authors:** Meiyun Wang, Xiubo Jiang, Wenlong Wu, Dongfeng Zhang

**Affiliations:** Department of Epidemiology and Health Statistics, Medical College of Qingdao University, Qingdao, Shandong, People's Republic of China; Kaohsiung Chang Gung Memorial Hospital, Taiwan

## Abstract

**Background:**

The association between polymorphism 4b/a, T-786C and G894T in endothelial NO synthase gene (eNOS) and ischemic stroke (IS) remains controversial in Asian. A meta-analysis was performed to better clarify the association between eNOS gene and IS risk.

**Methods:**

Based on the search of PubMed, Web of Science (ISI), CNKI (National Knowledge Infrastructure), Wan Fang Med Online and CBM (Chinese Biology Medical Literature Database) databases, all eligible case-control or cohort studies were identified. Pooled odds ratios (ORs) with 95% confidence intervals (CIs) from fixed and random effect models were calculated. Heterogeneity among studies was evaluated using the I^2^. Meta-regression was used to explore the potential sources of between-study heterogeneity. Begg's test was used to estimate publication bias.

**Results:**

Our study included 27 articles, contained 28 independent case–control studies, involved a total of 3,742 cases and 3,691 controls about 4b/a, 1,800 cases and 1,751 controls about T-786C and 2,747 cases and 2,872 controls about G894T. A significant association of 4a allele with increased risk of IS was found in dominant (FEM: OR = 1.498, 95% CI = 1.329–1.689), recessive (FEM: OR = 2.132, 95% CI = 1.383–3.286) and codominant (REM: OR = 1.456, 95% CI = 1.235–1.716) models. For T-786C and G894T, there were significant associations with dominant and codominant genetic models, but not with recessive genetic model.

**Conclusions:**

The meta-analysis indicated that eNOS gene 4b/a, T-786C, G894T polymorphism might be associated with IS.

## Introduction

Stroke is a major cause of morbidity and mortality worldwide [1], [2]. About 83% of strokes are ischemic stroke [3]. World Health Organization declared that about 5.5 million people died of stroke in 2002, and more than 50% happened in Asian countries such as China, Japan, Indian, Korea and so on [4].

Large epidemiological studies have shown that stroke has a genetic predisposition. Duggirala et al. thought that the proportion of genetic factors in the occurrence of stroke was about 66.0%–74.9% [5] and almost 80% of strokes are ischemic in origin [6]. Nitric oxide (NO), a pluripotent regulatory gas in the cerebrovascular system, may have an anti-thromboembolic effect by reducing both platelet adhesion [7] and aggregation [8]. NO is synthesized by the nitric oxide synthase (NOS) isoenzymes gene, of which three major NOS forms were described: endothelial (eNOS), neuronal (nNOS), and cytokine-inducible (iNOS). Studies suggest that eNOS is most likely to synthesize the NO that is responsible for maintaining resting cerebral blood flow [9]. The participation of the eNOS gene in the physiology of the vasculature makes it a biologically plausible candidate for study as a susceptibility gene for ischemic stroke [10].

The gene encoding eNOS is located on chromosome 7 (7q35–q36), spanning 21 kb and comprising 26 exons [11], [12]. In particular, three polymorphisms in eNOS have attracted much attention, namely 4b/a, T-786C and G894T. These variants were associated with vascular disorders, including stroke [13]. Studies have been conducted to evaluate the effect of eNOS gene (4b/a, T-786C, G894T) genetic polymorphisms on the risk of IS in Asian, however the results were conflicting. Hence, we perform the current meta-analysis to identify the association of eNOS gene and the risk of IS.

## Materials and Methods

### Literature Search

The available articles published in English or Chinese (up to July, 2012) were identified by extended computer-based searches from the following databases: (1) PubMed; (2) Web of Science; (3) CNKI (National Knowledge Infrastructure); (4) Wan Fang Med Online and (5) CBM (Chinese Biology Medical Literature Database). The following keywords were used: (‘eNOS’ or ‘endothelial nitric oxide synthase’ or ‘NOS3’) and (‘polymorphism’ or ‘mutation’ or ‘genes’) and (‘4b/a’or ‘T-786C’or ‘G894T’) and (‘ischemic stroke’ or ‘stroke’ or ‘brain infarction’ or ‘brain ischemia’ or ‘cerebrovascular disease’). We also reviewed the references cited in the studies and review articles to identify additional studies not captured by our database searches.

### Inclusion criteria

The inclusion criteria were as follows: (1) case-control or cohort study published as original study to evaluate the association between (4b/a, G894T and T-786C) polymorphisms in eNOS gene and risk of IS in Asian; (2) Neuroimaging (computed tomography (CT) or magnetic resonance imaging (MRI)) was used to confirm the diagnosis of IS; (3) numbers were reported in case and control groups for case-control studies, or exposed and unexposed groups for cohort studies for each genotype, or data provided from which numbers could be calculated; (4) subjects in each study should come from the same ethnicity and period; (5)Subjects>18 years age. The most recent and complete articles were chosen if one data from the same population had been published more than once. Two investigators carefully reviewed all identified studies independently to determine whether an individual study was eligible for inclusion criteria in this meta-analysis.

### Data extraction

Two investigators collected the data independently and reached a consensus on all items. The following basic information was extracted from the eligible studies: first author, journal, year of publication, country, ethnicity of studied population, sample size, mean age, male sex percentage, and distributions of allele and genotype. When it came to conflicting evaluations, it was resolved by the third reviewer.

### Statistical analysis

Departure from Hardy–Weinberg equilibrium (HWE) for the 4b/a, T-786C and G894T genotype distribution of eNOS gene in controls was tested by χ^2^ analysis with exact probability, (HWE: P >0.05). ORs with 95%CI was used to assess the strength of the association of the 4b/a, T-786C and G894T polymorphisms in eNOS gene with risk of IS. We conducted analysis for three polymorphisms considering dominant (aa + ba vs. bb), (CC + TC vs. TT), (TT + GT vs. GG); recessive (aa vs. ba + bb), (CC vs. TC+ TT), (TT vs. GT + GG) and codominant (a vs. b), (C vs. T), (T vs. G) models for 4b/a, T-786C and G894T respectively. I^2^ of Higgins and Thompson [14] was used to assess heterogeneity among studies. The DerSimo-nian and Laird random effect model (REM) was adopted as the pooling method if substantial heterogeneity is present (I^2^>50%) [15]; otherwise, the fixed effect model (FEM) was used as the pooling method. Meta regression with restricted maximum likelihood estimation was performed to explore the potentially important covariates: publication year, sample size (the sum of case numbers and control numbers), age (ratio of age or mean age in case group to that in control group), country (categorized as China and Non-China) and sex (ratio of male percent in case group to that in control group) that might exert substantial impacts on between-study heterogeneity. Influence analysis was conducted [16] to describe how robust the pooled estimator is to removal of individual studies. If the main estimate of an individual study's omitted analysis lies outside the 95% CI of the combined analysis, it is suspected of excessive influence. Modified Begg's test [17] was used to estimate publication bias. Subgroup analyses by country were conducted in Chinese population between the eNOS gene polymorphisms (4b/a, T-786C and G894T) and IS risk. Further investigation by Non-China population was not conducted for IS risk due to small number of studies included. Besides, both theoretical and empirical evidence suggest that general genetic variants causally associated with common diseases will have small effects (risk ratios mostly<2.0) [18], [19], and considering the fact that original studies with relatively small participants might be underpowered to detect the effect. Thus, for sensitivity analysis, we further excluded the studies with OR>3.0 as the criteria to control the impact within each single study on the pooled effect. STATA version 10.0 (Stata Corporation, College Station, TX, USA) was used to perform statistical analyses. All reported probabilities (p values) were two-sided, with p-value less than 0.05 considered representative of statistically significant.

## Results

### Characteristics of studies

**Figure 1:**
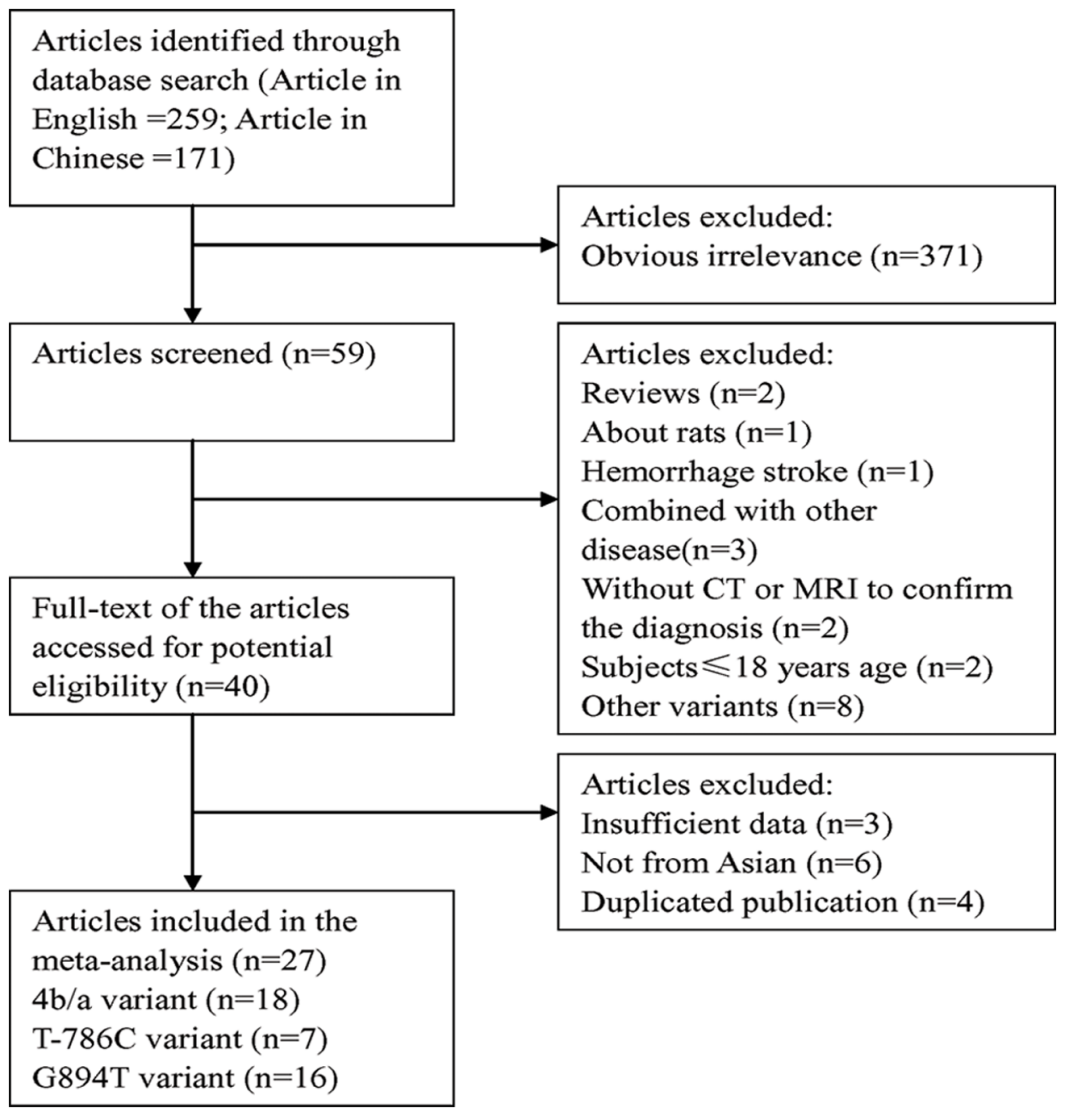
Flow chart of meta-analysis for exclusion/inclusion of studies.

We identified 27 articles [13], [20]–[45] with 28 eligible outcomes for this meta-analysis, including 18 outcomes for 4b/a polymorphism; 7 outcomes for T-786C polymorphism and 16 outcomes for G894T polymorphism. All 27 articles were case-control designs. General characteristics and genotype distributions of the above-mentioned polymorphisms are summarized in Tables 1–3. Figure 1. presented the flow chart for exclusion/inclusion process. The details of reasons for exclusion of studies from meta-analysis are listed in Supplementary Table S1.

**Table 1:** Characteristics of eNOS gene 4b/a polymorphism genotype distributions in studies included in this meta-analysis.

Author	Year	Country	Number	Genotype bb/ba/aa		% of male	P for HWE
			(case/control)	case control		(case/control)	in control
Hou et al.	2001	China	364/516	263/77/24	439/71/6	221/313	0.13
Song et al.	2005	China	112/104	82/28/2	86/17/1	65/56	0.60
Zhang et al.	2007	China	488/420	387/90/11	359/57/4	298/234	0.30
Zhang et al.	2004	China	152/114	116/34/2	98/14/2	88/66	0.14
Munshi et al.	2010	Indian	357/283	172/146/39	181/89/13	264/210	0.59
Pu et al.	2003	China	170/90	134/28/8	77/9/4	92/46	0.00 *
Yemisci et al.	2009	Turkey	70/81	49/20/1	44/24/13	44/33	0.01 *
Wang et al.	2007	China	58/60	45/11/2	45/10/5	34/32	0.01 *
Wang et al.	2007	China	58/60	45/11/2	48/10/2	35/30	0.16
Kim et al.	2010	Korean	156/170	129/27/0	144/26/0	92/84	0.60
Luo et al.	2011	China	148/130	112/29/7	113/16/1	85/78	0.47
Zhou et al.	2009	China	468/596	368/87/13	508/81/7	338/489	0.09
Song et al.	2010	Korean	269/234	214/43/8	181/41/0	124/116	0.23
Yahashi et al.	1998	Japan	127/91	93/33/1	67/24/0	62/49	0.35
Fang et al.	2011	China	195/237	157/33/0	187/48/1	150190	0.49
Han et al.	2010	China	112/105	85/25/2	91/13/1	62/53	0.42
li et al.	2011	China	300/300	236/54/10	247/50/3	192/192	0.73
Meng et al.	2012	China	138/100	121/15/0	84/16/0	99/64	1.00

* Studies deviated from the Hardy-Weinberg equilibrium

**Table 2 pone-0060472-t002:** Characteristics of eNOS gene T-786C polymorphism genotype distributions in studies included in this meta-analysis.

Author	Year	Country	Number	Genotype TT/TC/CC		% of male	P for HWE
			(case/control)	case control		(case/control)	in control
Cheng et al.	2008	China	309/309	240/58/11	250/56/3	190/190	1.00
Yemisci et al.	2009	Turkey	70/81	25/33/9	35/41/3	44/38	0.06
Kim et al.	2010	Korean	156/170	129/27/0	145/25/0	92/84	0.60
Yan et al.	2011	China	558/557	434/122/2	451/96/10	361/346	0.11
Song et al.	2010	Korean	269/234	218/43/4	193/39/0	124/116	0.38
li et al.	2011	China	300/300	230/66/4	238/57/5	192/192	0.39
Meng et al.	2012	China	138/100	124/14/0	90/10/0	99/64	1.00

**Table 3:** Characteristics of eNOS gene G894T polymorphism genotype distributions in studies included in this meta-analysis.

Author	Year	Country	Number	Genotype GG/GT/TT		% of male	P for HWE
			(case/control)	case control		(case/control)	in control
Lv et al.	2004	China	100/100	69/26/5	84/14/2	62/62	0.17
Cheng et al.	2008	China	309/309	235/70/4	243/61/5	61/61	0.57
Cui et al.	2007	China	114/76	80/25/9	63/12/1	61/58	0.48
Majumdar et al.	2010	Indian	172/214	124/43/5	159/50/5	69/67	0.58
Wang et al.	2007	China	58/60	47/9/2	49/10/1	60/50	0.46
Wang et al.	2007	China	58/60	51/5/2	46/13/1	59/53	1.00
Zhang et al.	2009	China	132/128	107/22/3	116/12/0	55/55	1.00
Xu et al.	2004	China	148/109	123/21/4/	99/10/0	60/83	1.00
Yan et al.	2011	China	545/557	417/123/5	446/102/9	65/62	0.27
Guldiken et al.	2008	Turkey	146/133	82/59/5	66/61/6	51/26	0.13
Luo et al.	2011	China	148/130	118/29/1	115/15/0	57/60	1.00
Song et al.	2010	Korean	265/230	185/78/2	190/40/0	46/50	0.23
Yemisci et al.	2009	Turkey	67/81	23/36/8	22/47/12	63/47	0.17
Li et al.	2011	China	300/300	235/56/9	245/53/2	64/64	1.00
Di et al.	2004	China	40/169	27/13/0	139/30/0	Na/Na	0.37
Moe et al.	2008	Singapore	118/207	89/26/3	160/42/5	76/69	0.33

Na: not available.

### Quantitative synthesis

The results of the pooled analysis are summarized in Table 4.

**Table 4:** Pooled measures on the relationship of eNOS gene 4b/a, T-786C and G894T polymorphism with ischemic stroke.

			All included articles				After excluding for DHWE and articles with OR>3.0				
Polymorphism		Inherited	Pooled OR (95% CI)		I^2^	P^c^	Pooled OR (95% CI)		I^2^	P^c^	Articles
		model	FEM	REM	(%)	Value	FEM	REM	(%)	Value	excluded
All	4b/a	Dominant	1.439(1.283–1.615)	1.350(1.125–1.620)	54.7	0.001	1.498(1.329–1.689)	1.439(1.209–1.714)	47.1	0.000	[24], [35], [42]
data		Recessive^a^	2.176(1.552–3.052)	1.853(1.112–3.087)	43.4	0.000	2.132(1.383–3.286)	2.132(1.383–3.286)	0.0	0.001	[13], [24], [35], [40]–[43]
		Codominant	1.447(1.306–1.604)	1.310(1.081–1.586)	67.1	0.006	1.537(1.381–1.712)	1.456(1.235–1.716)	50.9	0.000	[24], [35], [42]
	T-786C	Dominant	1.186(1.004–1.401)	1.186(1.004–1.401)	0.0	0.044	_	_	_	_	_
		Recessive^a^	1.529(0.787–2.969)	1.576(0.455–5.460)	69.0	0.473	*				[32], [41], [42]
		Codominant	1.183(1.018–1.375)	1.183(1.018–1.375)	0.0	0.029	_	_	_	_	_
	G894T	Dominant	1.304(1.146–1.485)	1.336(1.101–1.621)	48.2	0.000	_	_	_	_	_
		Recessive^a^	1.273(0.848–1.910)	1.273(0.848–1.910)	0.00	0.245	0.959(0.615–1.495)	0.959(0.615–1.495)	0.0	0.854	[33], [36], [37], [41], [43]
		Codominant	1.269(1.131–1.424)	1.336(1.112–1.604)	54.6	0.002	_	_	_	_	_
China	4b/a	Dominant	1.511(1.317–1.733)	1.463(1.202–1.781)	43.7	0.000	1.532(1.329–1.765)	1.489(1.204–1.843)	49.0	0.000	[24], [35]
		Recessive^b^	2.244(1.488–3.385)	2.067(1.258–3.397)	23.4	0.000	1.838(1.018–3.319)	1.838(1.018–3.319)	0.0	0.043	[13], [24], [35], [40], [43]
		Codominant	1.526(1.347–1.728)	1.432(1.169–1.753)	55.9	0.001	1.566(1.376–1.783)	1.490(1.204–1.842)	56.9	0.000	[24], [35]
	T-786C	Dominant	1.193(0.982–1.449)	1.193(0.982–1.449)	0.0	0.075	_	_	_	_	_
		Recessive^b^	0.982(0.446–2.162)	0.872(0.169–4.511)	76.7	0.871	*				[32]
		Codominant	1.154(0.966–1.379)	1.154(0.966–1.379)	0.0	0.115	_	_	_	_	_
	G894T	Dominant	1.390(1.184–1.631)	1.463(1.173–1.825)	36.1	0.000	_	_	_	_	_
		Recessive^b^	1.683(0.951–2.976)	1.834(0.972–3.460)	13.4	0.074	1.040(0.530–2.043)	1.040(0.530–2.043)	0.0	0.909	[33], [36], [37], [43]
		Codominant	1.380(1.193–1.598)	1.494(1.199–1.861)	45.2	0.000	_	_	_	_	_

DHWE: deviated from Hardy–Weinberg equilibrium in controls.

a Two articles (Kim et al. Meng et al.) in 4b/a and T-786C and one article (Di et al.) in G894T for recessive model were not sufficient to calculated pooled OR.

b One article (Meng et al.) in 4b/a and T-786C and one article (Di et al.) in G894T for recessive model were not sufficient to calculated pooled OR.

c p-values was for observed pooled odds ratios. when I^2^≤50%, it was for FEM, otherwise it was for REM.

* Pooled ORs were not calculated for only two articles left sufficient to calculate pooled ORs after excluding articles with OR>3.0.

FEM, fixed effect model; REM, random effect model

Dominant model: aa + ba vs. bb for 4b/a, CC + TC vs. TT for T-786C, and TT + GT vs. GG for G894T.

Recessive model: aa vs. ba + bb for 4b/a, CC vs. TC + TT for T-786C, and TT vs. GT + GG for G894T.

Codominant model: a vs. b for 4b/a, C vs. T for T-786C, and for T vs. G for G894T.

### The 4b/a polymorphism

**Figure 2:**
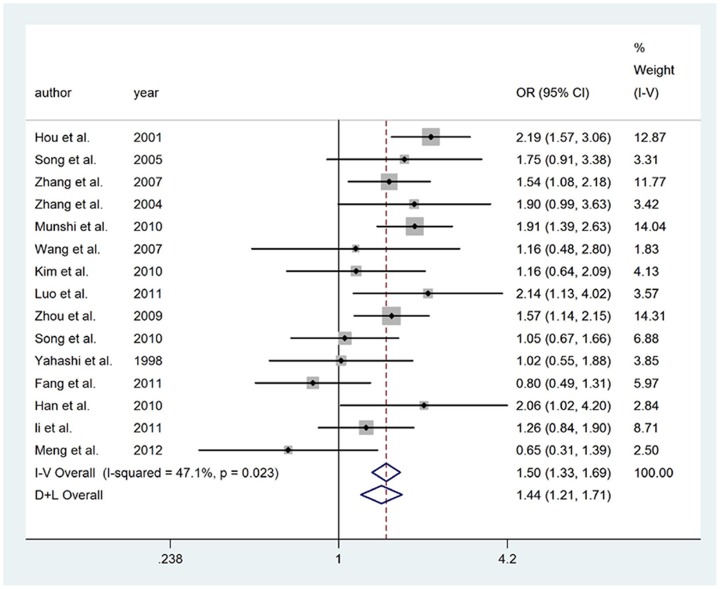
Meta-analysis for IS risk depending on the 4b/a polymorphism in the eNOS gene. Forest plots of relationship between eNOS gene 4b/a polymorphism and IS risk in dominant model after excluding articles that that deviated from HWE in controls in Asian. White diamond denotes the pooled OR. Black squares indicate the OR in each study, with square sizes inversely proportional to the standard error of the OR. Horizontal lines represent 95% CIs.

For 4b/a polymorphism, overall, significant association was found between the 4a allele and IS risk in dominant (REM: OR = 1.350, 95%CI = 1.125–1.620), recessive (FEM: OR = 2.176, 95%CI = 1.552–3.052) and codominant (REM: OR = 1.310, 95%CI = 1.081–1.586) models. In subgroup analysis, for Chinese population, the 4a allele was found contributing significantly to increased IS risk in dominant (FEM: OR = 1.511, 95%CI = 1.317–1.733), recessive (FEM: OR = 2.244, 95%CI = 1.488–3.385) and codominant (FEM: OR = 1.526, 95%CI = 1.347–1.728) models. All the associations were not altered significantly after excluding articles [24], [35], [42] deviating from HWE in controls. Figure 2. presented the forest plot of ORs for IS in dominant model of eNOS gene 4b/a polymorphism in Asian after excluding articles deviating from HWE in controls.

### The T-786C polymorphism

**Figure 3:**
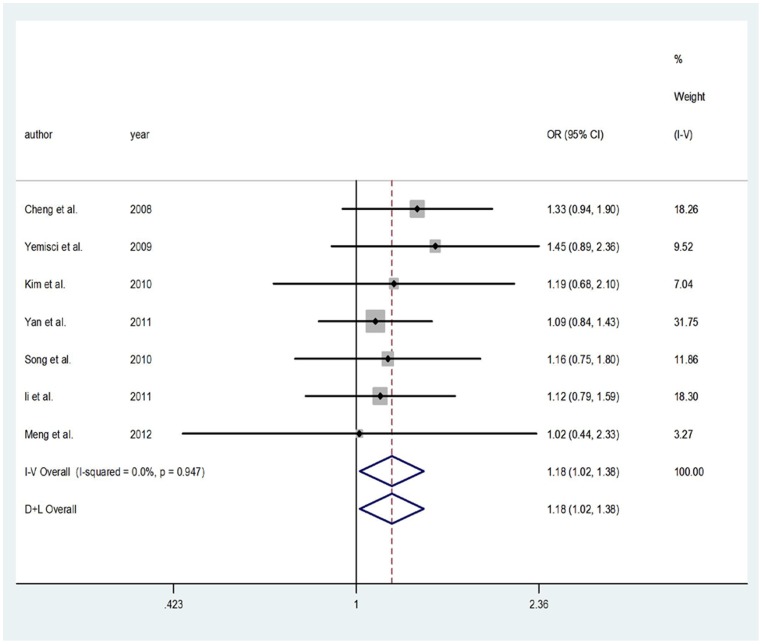
Meta-analysis for IS risk depending on the T-786C polymorphism in the eNOS gene. Forest plots of relationship between eNOS gene T-786C polymorphism and IS risk in codominant model in Asian. White diamond denotes the pooled OR. Black squares indicate the OR in each study, with square sizes inversely proportional to the standard error of the OR. Horizontal lines represent 95% CIs.

For T-786C polymorphism, this meta-analysis showed a significant protective effect of the C allele on IS risk in dominant (FEM: OR = 1.186, 95%CI = 1.004–1.401), codominant (FEM: OR = 1.183, 95%CI = 1.018–1.375) models, but not in recessive (REM: OR = 1.576, 95%CI = 0.455–5.460) model. In subgroup analysis by country, no significant association was found in any of the above-mentioned inherited models for Chinese population. All included articles were in HWE in controls for T-786C. Figure 3. presented the forest plot of ORs for IS in codominant model of eNOS gene T-786C polymorphism in Asian.

### The G894T polymorphism

**Figure 4:**
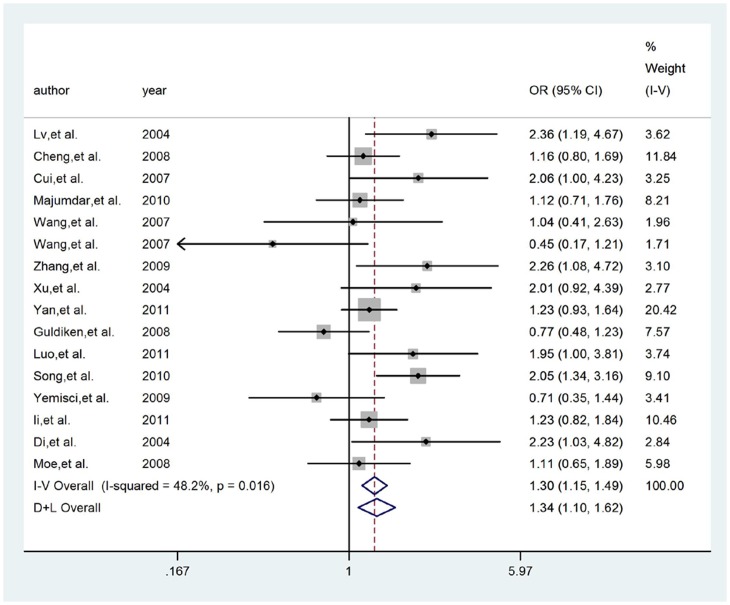
Meta-analysis for IS risk depending on the G894T polymorphism in the eNOS gene. Forest plots of relationship between eNOS gene G894T polymorphism and IS risk in dominant model in Asian. White diamond denotes the pooled OR. Black squares indicate the OR in each study, with square sizes inversely proportional to the standard error of the OR. Horizontal lines represent 95% CIs.

For G894T polymorphism, overall, significant association was found between T allele and IS risk in dominant (FEM: OR = 1.304, 95%CI = 1.146–1.485) and codominant (REM: OR = 1.336, 95%CI = 1.112–1.604) models, but not in recessive (FEM: OR = 1.273, 95%CI = 0.848–1.910) model. And subgroup analysis, for Chinese population, significant association was found in dominant (FEM: OR = 1.390, 95%CI = 1.184–1.631) and codominant (FEM: OR = 1.380, 95%CI = 1.193–1.598) models, but not in recessive (FEM: OR = 1.683, 95%CI = 0.951–2.976) model. All included articles were in HWE in controls. Figure 4. presented the forest plot of ORs for IS in dominant model of eNOS gene G894T polymorphism in Asian.

### Sensitivity analysis after excluding articles deviating from HWE in controls

#### The 4b/a polymorphism

For IS risk in overall, after exclusion of articles with OR>3.0 [13], [40], [41], [43], the risk effects of the 4a allele in the recessive model (FEM: OR = 2.132, 95%CI = 1.383–3.286) remained significant. No studies with OR>3.0 existed in dominant and codominant models. And the results for Chinese comparison groups remained significant.

#### The T-786C polymorphism

If the frequency of mutation homozygous were 0 in case group, it was not sufficient to calculate pooled OR for recessive model. For this reason, in T-786C polymorphism, two articles [29], [30] for recessive model were not sufficient to calculated pooled OR. After excluding articles for OR>3.0 [32], [41], [42], there were only two studies left sufficient to calculated pooled OR for recessive model, so we did not further investigate the association in recessive model. No studies with OR>3.0 existed in dominant and codominant models.

#### The G894T polymorphism

After excluding articles for OR>3.0 [33], [36], [37], [41], [43], the association of the T allele of the G894T polymorphism with IS in recessive model remained no significant (FEM: OR = 0.959, 95%CI = 0.615–1.495). The associations in Chinese comparison groups remained non-significant for recessive model. No studies with OR>3.0 existed in dominant and codominant models.

### Sources of heterogeneity

After exclusion of articles deviating from HWE in controls, evidence for heterogeneity (I^2^>50%) was found in the codominant model considering the association of 4b/a polymorphism with IS risk; T-786C polymorphism in recessive model and G894T in codominant model, respectively.

Univariate meta-regression with the covariates of publication year, sample size, age, country and sex for the above-mentioned polymorphisms, showed that no covariates had a significant impact on between-study heterogeneity.

### Influence analysis

After exclusion of articles deviating from HWE in controls and sensitivity analysis, no individual study was found having excessive influence on the pooled effect in any of dominant, recessive and codominant models considering all the polymorphisms (data not shown).

### Publication bias

Begg's test was used to assess the publication bias. After exclusion of articles deviating from HWE in controls and sensitivity analysis, no significant publication bias was detected in any of the above-mentioned inherited models considering all the polymorphisms except the recessive model in G894T polymorphism (data not shown).

## Discussion

Ischemic stroke is known to be a multifactorial disorder. In addition to the commonly accepted risk factors, there is increasing evidence for the role of genes in the pathophysiology of ischemic stroke [46]. NO synthesized by the nitric oxide synthase (NOS) is a pluripotent regulatory gas in the cardiovascular system. eNOS-derived NO plays an important role in the maintenance of vascular homeostasis, including regulation of the cerebral circulation [10]. Besides, previous studies suggested that eNOS gene (4b/a,T-786C,G894T) polymorphisms are significantly associated with hypertension [47], [48], total cholesterol and low-density lipoprotein cholesterol levels [49], [50].

Recently, studies have been performed to evaluate the correlation between eNOS gene (4b/a,T-786C,G894T) polymorphisms and the risk of IS. However, the results remained controversial. Since an individual study has a relatively small number of participants with low power to detect the effect, a meta-analysis may be the appropriate approach to obtain a more definitive conclusion. In this meta-analysis, we summarize the results of 27 case-control articles with 28 eligible outcomes published so far on the association between eNOS gene (4b/a,T-786C,G894T) polymorphisms and IS risk. A significant association of 4a allele with increased risk of IS was found in dominant, recessive and codominant models. For T-786C and G894T, there were significant associations with dominant and codominant genetic models, but not with recessive genetic model. In the subgroups, for Chinese population, the results were consistent with overall results. The results of our study were contrary with the previous meta-analysis in 2009 [10]. The reasons might be that the numbers of studies adopted in previous meta-analysis were relatively small and the genetic differentiations varying with ethnic.

For a simple genetic variant with two alleles, the classical models of inheritance (dominant, recessive and codominant model) are typically assumed for complex traits when the inheritance model is unknown [51], [52]. Besides, maximal power is achieved, when the inherited model is unknown, with codominant model alone or all three genetic models tested together [53]. Thus we tested all three models simultaneously. To evaluate gene–disease associations, there is rarely a priori biologic evidence supporting a particular genetic model of inheritance for the risk allele. However, in the setting of an agnostic approach, most associations derived from genome-wide association studies are also usually presented as per-allele risks (the codominant model) [54] because of statistical power considerations.

Between-study heterogeneity is common in meta-analysis for genetic association studies [55], and exploring the potential sources of between-study heterogeneity is the essential component of meta-analysis [56]. For 4b/a and G894T, moderate to high heterogeneity was found in dominant and codominant models, but no heterogeneity exists in recessive model after excluding articles that deviated from HWE in controls and sensitivity analysis. For T-786C, high heterogeneity was found in recessive mode but no heterogeneity exists in dominant and codominant models. The between-study heterogeneity might arise from an indeterminate number of characteristics that vary among studies. The possibilities related to the disease-effect diversity, such as study quality, characteristics of the subjects, genotyping, clinical heterogeneity (diagnosis for IS patients, allelic or locus) and lifestyle factors etc., could not be ruled out, even though no significant individual study influence on the pooled effect was observed with influence analysis. Thus we used meta-regression to explore the causes of heterogeneity for covariates. However, publication year, mean age, country and sex were not found to be important sources of disease–effect heterogeneity in this meta-analysis. Considering meta-analysis of all included studies was fraught with the problem of heterogeneity. Subgroup analyses only included Chinese population was performed to explore the source of heterogeneity. However, the between-study heterogeneity persisted in some genetic models suggesting the presence of other unknown confounding factors. IS have a complex aetiology and pathophysiology generated by the combined effects of genes and environment factors. Thus other genetic and environment variables, as well as their possible interaction, may well be potential contributors to the heterogeneity observed.

In this meta-analysis, no significant publication bias for 4b/a, G894T polymorphisms in any of the above-mentioned inherited models, suggesting the associations observed should be stable.

In conclusion, our meta-analysis suggested that eNOS gene 4b/a, T-786C, G894T polymorphism might be associated with IS. This conclusion highlighted the importance of the eNOS gene on IS in Asian which might be a useful suggestion for clinical therapeutic intervention. The present study is limited by we could not completely preclude the potential biases and confounders in meta-analysis. Further research is warranted to confirm our findings.

## Supporting Information

Table S1 **Details of reasons for exclusion of studies from meta-analysis.**
(DOC)

Table S2 **PRISMA checklist.**
(DOC)
