# Supplementary material for: Endothelial NO Synthase Gene Polymorphisms and Risk of Ischemic Stroke in Asian Population: A Meta-Analysis
Source: PLoS One. 2013 Mar 27;8(3):e60472. doi: 10.1371/journal.pone.0060472 (PMC3609746; doi:10.1371/journal.pone.0060472)
Supplement: Table S1 — Details of reasons for exclusion of studies from meta-analysis. (DOC) [file pone.0060472.s001.doc]

Supplementary table S1: Details of reasons for exclusion of studies from meta-analysis

| References | Reason for exclusion |
| --- | --- |
| [1, 2] | Reviews |
| [3] | About rats |
| [4] | Hemorrhage stroke |
| [5-7] | Combined with other disease |
| [8, 9] | Without CT or MRI to confirm the diagnosis |
| [10, 11] | Subjects≤18 years age |
| [12-19] | Other variants |
| [20-22] | Insufficient data |
| [23-28] | Not from Asian |
| [29-32] | Duplicated publication |

**References**

1.Sawada, N,JK Liao (2009) Targeting eNOS and beyond: emerging heterogeneity of the role of endothelial Rho proteins in stroke protection. Expert Rev Neurother 9:1171-86.

2.Rao, R, V Tah, JP Casas, A Hingorani, J Whittaker, et al. (2009) Ischaemic stroke subtypes and their genetic basis: a comprehensive meta-analysis of small and large vessel stroke. Eur Neurol 61:76-86.

3.Son, HY, HW Jung, WK Kim,YK Park (2010) The vasoprotective effect of JP05 through the activation of PI3K/Akt-dependent eNOS and MEK/ERK pathways in brain endothelial cells. J Ethnopharmacol 130:607-13.

4.Ko, NU, P Rajendran, H Kim, M Rutkowski, L Pawlikowska, et al. (2008) Endothelial nitric oxide synthase polymorphism (-786T->C) and increased risk of angiographic vasospasm after aneurysmal subarachnoid hemorrhage. Stroke 39:1103-8.

5.Ge, RL, FM Chen, XF Han,CH Su (2005) Research about the a/b in intron 4 polymorphism of endothelial nitric synthase(eNOS) gene in type 2 diabetea with cerebral infarction. Journal of Binzhou Meical College 28:408-410.

6.Ge, RL, FM Chen, JB Chen, XF Han,XS Zhou (2011) Association between polymorphism of endothelial nitric synthase gene in type 2 diabetea with cerebral infarction in aged. BMU Journal 34:16-18.

7.Zhao, YX,QH Lu (2006) Association of the endothelial nitric synthase gene polymorphism with essentional hypertension and its cardiocerebrovascular complications. Shandong University Masters Thesis

8.Howard, TD, WH Giles, J Xu, MA Wozniak, AM Malarcher, et al. (2005) Promoter polymorphisms in the nitric oxide synthase 3 gene are associated with ischemic stroke susceptibility in young black women. Stroke 36:1848-51.

9.Zhao, Y, LY Ma, LS Liu, YX LIU, XY Wang, et al. (2000) Relationship between eNOS gene G894T mutation and ischemic stroke. Chin J Crit Care Med 20:457-458.

10.Shi, C, X Kang, Y Wang,Y Zhou (2008) The coagulation factor V Leiden, MTHFRC677T variant and eNOS 4ab polymorphism in young Chinese population with ischemic stroke. Clin Chim Acta 396:7-9.

11.Djordjevic, V, M Stankovic, V Brankovic-Sreckovic, L Rakicevic,D Radojkovic (2009) Genetic risk factors for arterial ischemic stroke in children: a possible MTHFR and eNOS gene-gene interplay? J Child Neurol 24:823-7.

12.Du, D, P Gao, L Hu, Y Yang, F Wang, et al. (2008) A genetic study of the NOS3 gene for ischemic stroke in a Chinese population. Int J Gen Med 1:65-8.

13.Lin, NT, MJ Lee, RP Lee, AI Hong,HI Chen (2008) Analysis of endothelial nitric oxide synthase gene polymorphisms with cardiovascular diseases in eastern Taiwan. Chin J Physiol 51:42-7.

14.Suzuki, S, A Kurata, M Yamada, K Iwamoto, K Nakahara, et al. (2011) Contrast stasis in large and giant internal carotid artery aneurysms as a good prognostic factor for endovascular coil embolization: retrospective study. Neurol Res 33:832-4.

15.Oksala, NK, A Oksala, T Erkinjuntti, T Pohjasvaara, T Kunnas, et al. (2008) Long-term survival after ischemic stroke in postmenopausal women is affected by an interaction between smoking and genetic variation in nitric oxide synthases. Cerebrovasc Dis 26:250-8.

16.Cheng, JQ, JP Liu, RL Zhang, L Yu, J Peng, et al. (2008) Study on A-922G genetic polymorphism of endothelial nitric synthase associated ischemic stroke. JOURNAL OF HYGIENE RESEARCH 37:1-3.

17.Manso, H, T Krug, J Sobral, I Albergaria, G Gaspar, et al. (2012) Variants within the nitric oxide synthase 1 gene are associated with stroke susceptibility. Atherosclerosis 220:443-8.

18.Greco, R, AS Mangione, D Amantea, G Bagetta, G Nappi, et al. (2011) IkappaB-alpha expression following transient focal cerebral ischemia is modulated by nitric oxide. Brain Res 1372:145-51.

19.Gao, P, J Wu, DH DU, JX ZHAO, LS Hu, et al. (2007) Association study between a polymorphism of endothelial nitric oxide synthase gene and ischemic stroke. J Apoplexy and Nervous Diseases 24:644-646.

20.M.J. MacLeod, M.T. Dahiyat, A. Cumming, D. Meiklejohn, D. Shaw, et al. No association between Glu/Asp polymorphism of NOS3 gene and ischemic stroke.

21.Ma, X, JP Jia, XM Dong,M Wen (2007) Association of nitric Oxide Synthase Gene and Nitric Oxide Production in Cerebrovascular Infarction. Chin J Rehabil Theory Pract 13:845-847.

22.Dutra, AV, HF Lin, SH Juo, M Boyadjis, M Moussouttas, et al. (2006) Analysis of the endothelial nitric oxide synthase gene as a modifier of the cerebral response to ischemia. J Stroke Cerebrovasc Dis 15:128-31.

23.Saidi, S, SG Mallat, WY Almawi,T Mahjoub (2010) Endothelial nitric oxide synthase Glu298Asp, 4b/a, and -786T>C gene polymorphisms and the risk of ischemic stroke. Acta Neurol Scand 121:114-9.

24.Elbaz, A, O Poirier, T Moulin, F Chedru, F Cambien, et al. (2000) Association between the Glu298Asp polymorphism in the endothelial constitutive nitric oxide synthase gene and brain infarction. The GENIC Investigators. Stroke 31:1634-9.

25.Markus, HS, Y Ruigrok, N Ali,JF Powell (1998) Endothelial nitric oxide synthase exon 7 polymorphism, ischemic cerebrovascular disease, and carotid atheroma. Stroke 29:1908-11.

26.Szolnoki, Z, V Havasi, J Bene, K Komlosi, D Szoke, et al. (2005) Endothelial nitric oxide synthase gene interactions and the risk of ischaemic stroke. Acta Neurol Scand 111:29-33.

27.Berger, K, F Stogbauer, M Stoll, J Wellmann, A Huge, et al. (2007) The glu298asp polymorphism in the nitric oxide synthase 3 gene is associated with the risk of ischemic stroke in two large independent case-control studies. Hum Genet 121:169-78.

28.Hassan, A, K Gormley, M O'Sullivan, J Knight, P Sham, et al. (2004) Endothelial nitric oxide gene haplotypes and risk of cerebral small-vessel disease. Stroke 35:654-9.

29.Ge, RL, FM Chen, DH Tian, CM Yang,C Zhang (2004) Association between variation number of tandem repeat polymorphism of endothelial nitric oxide synthase gene and cerebrovascular ischemic. J Clin Neurol 17:88.

30.Luo, N (2006) The association of endothelial nitric oxide synthase G894T in exon7 and in intron 4 gene polymorphisms with cerebrovascular infarction. Journal of Guangzhou Meical College

31.Ge, RL, FM Chen, XF Han, CH Su,H Ma (2005) Association study between polymorphisms of endothelial nitric oxide synthase gene and ischemic stroke with type 2 diabetes. Chin J Nerv Ment Dis 31:391-392.

32.Gao, HY,HX Han (2009) VNTR polymorphism of eNOS gene in elderly patients with ischemic stroke. Geriatr Health Care 15:345-347.
